# Disseminated Culture‐Negative Periprosthetic Knee Infection With Multifocal Septic Arthritis Associated With *Mycoplasma pneumoniae:* A Case Report

**DOI:** 10.1155/cro/9426714

**Published:** 2026-07-15

**Authors:** Brett Barthman, Matthew Klassen, Adrian Ressing, Kristopher Danielson

**Affiliations:** ^1^ Anatomy Department, A T Still University Kirksville College of Osteopathic Medicine, Kirksville, Missouri, USA; ^2^ Department of Orthopedics, University of Michigan Health-West, Wyoming, Michigan, USA

## Abstract

We report a case of a 72‐year‐old woman with Waldenström macroglobulinemia who developed a culture‐negative periprosthetic joint infection (PJI) of the right knee following total knee arthroplasty. Despite multiple debridements and broad‐spectrum antibiotics, she developed systemic signs of infection and hematogenous spread to multiple native joints, including the contralateral knee, ankle, wrist, and lumbar facet joints. All intraoperative cultures remained negative. A respiratory PCR was performed, which detected *Mycoplasma pneumoniae*, and metagenomic next‐generation sequencing (mNGS) of plasma supported the diagnosis of the pathogen. Based on these findings, therapy was narrowed to doxycycline, resulting in clinical improvement and deferral of further surgery. This case highlights the importance of considering atypical pathogens in culture‐negative PJI and demonstrates the utility of mNGS in guiding targeted antimicrobial therapy.

## 1. Introduction

Periprosthetic joint infection (PJI) is a complication of total joint arthroplasty that affects approximately 1%–2% of primary procedures and up to 4% of revision surgeries, despite advances in surgical technique and perioperative care [1, 2]. These infections are associated with significant morbidity, prolonged hospital stays, repeated surgeries, and increased mortality, particularly in cases complicated by bacteremia or sepsis [1, 3].

The majority of PJIs are caused by gram‐positive organisms such as *Staphylococcus aureus* and coagulase‐negative staphylococci [4]. However, atypical and fastidious microorganisms, such as *Mycoplasma hominis* and *Mycoplasma pneumoniae*, may be underrecognized contributors in selected populations. Although *M. hominis* has been associated with genitourinary and periprosthetic infections, *M. pneumoniae* is typically a respiratory pathogen. Rare extrapulmonary manifestations have been reported with 34 documented cases of septic arthritis from *M. pneumoniae*, of which eight were PJIs [5]. Its prevalence in facet joint involvement has not been described in the current literature [5, 6]. Infectious Diseases Society of America (IDSA) states in their updated 2024 guidance the importance of early joint aspiration, targeted cultures, and the selective use of molecular diagnostics to aid in the accurate identification of pathogens in both acute and chronic presentations of PJI [3].

Although PJI typically presents as a localized infection confined to the prosthetic joint, hematogenous spread from a single prosthetic infection to multiple native joints is exceedingly rare [7, 8]. This presentation often mimics primary polyarticular septic arthritis and may delay diagnosis or appropriate source control [3, 4, 9]. In immunocompetent patients, such disseminated spread underscores the virulence of specific pathogens and the systemic inflammatory burden that can accompany untreated PJI.

Here, we present a case of a woman with an infected right knee prosthesis who developed sepsis with secondary involvement of multiple native joints, including the contralateral knee, right ankle, right wrist, and possibly left shoulder. This case highlights the potential for hematogenous dissemination from a single‐site periprosthetic joint infection (PJI), the importance of comprehensive joint evaluation in septic patients, and the need for multidisciplinary coordination in managing complex musculoskeletal infections.

## 2. Case Presentation

### 2.1. Initial Presentation and Index Procedure

A 72‐year‐old woman with Waldenström macroglobulinemia, chronic anemia, hypertension, glaucoma, and GERD underwent right total knee arthroplasty (TKA) on February 21, 2025. She was admitted on April 26 for right knee pain and drainage; synovial fluid analysis showed 110,000 nucleated cells (81% neutrophils), and she underwent irrigation and debridement (I&D) with polyethylene exchange. PJI was diagnosed using the 2018 International Consensus Meeting (ICM) criteria, which include elevated synovial leukocyte count, neutrophil predominance, purulence at surgery, and clinical signs of infection. All cultures remained negative. She was discharged on May 1 with intravenous ceftriaxone and daptomycin.

### 2.2. Readmission and Diagnostic Evaluation

Over the next several weeks in rehabilitation, she experienced persistent right knee pain, intermittent left knee and shoulder pain, and episodes of confusion. On May 25, she developed worsening confusion, bowel and bladder incontinence, and leukocytosis (14.1 × 10^9^/L). She was admitted the next day; outpatient antibiotics were discontinued, and empiric meropenem and vancomycin were initiated. Although she did not report prominent respiratory symptoms, a respiratory pathogen PCR (nasopharyngeal swab) was obtained as part of a broad infectious evaluation for unexplained systemic illness and encephalopathy, and was positive for *M. pneumoniae*. Testing was performed on a nasopharyngeal swab. Aspiration of the right prosthetic knee yielded 220 nucleated cells (98% neutrophils). The markedly lower synovial leukocyte count compared with the initial aspiration (110,000 cells/*μ*L) was attributed to several weeks of ongoing intravenous antibiotic therapy prior to repeat aspiration, which is known to significantly reduce synovial leukocyte counts despite persistent infection. Table 1 summarizes the diagnostic and therapeutic timeline, including initial empiric antimicrobial therapy, surgical interventions, molecular diagnostic testing, and subsequent targeted treatment.

**Table 1:** Summary of antimicrobial therapy during hospitalization and follow‐up.

Date range	Antibiotic(s)	Dose and route	Indication/rationale	Reason for change
4/26–5/1	Ceftriaxone + daptomycin	2 g IV q24h + 10 mg/kg IV q24h	Empiric coverage after the first right knee I&D with poly exchange	Discharge to SAR; continued outpatient regimen
5/1–5/26	Ceftriaxone + daptomycin	Same as above	Continued outpatient therapy for suspected PJI	Clinical deterioration with knee drainage and confusion prompted hospital readmission
5/26–5/27	Meropenem + vancomycin	2 g IV q8h + weight − based dosing IV	Broad‐spectrum empiric coverage for possible multidrug‐resistant organisms	Adjustment after ID consult
5/27–6/1	Meropenem + vancomycin + doxycycline	Same as above + 100 mg PO/IV BID	Added for possible atypical pneumonia (*M. pneumoniae*) after positive respiratory PCR	Completed planned 5‐day doxycycline course
6/2–6/7	Ceftriaxone + vancomycin	2 g IV q24h + weight − based dosing IV	Narrowed empiric coverage postsurgery	MRI findings and ongoing multifocal infections prompted readdition of doxycycline
6/7–6/10	Ceftriaxone + vancomycin + doxycycline	Same as above	Coverage pending molecular test results (mNGS)	Positive mNGS for *M. pneumoniae* → narrowed regimen
6/10–7/24	Doxycycline	100 mg IV/PO BID	Targeted therapy for *M. pneumoniae*	Completed until revision TKA
7/24–7/28	Daptomycin + ceftriaxone	10 mg/kg IV q24h + 2 g IV q24h + 100 mg BID	Postrevision empiric broad‐spectrum coverage	Transitioned due to prolonged antibiotic exposure
7/28–9/8	Ertapenem	1 g IV q24h	Planned 6‐week course postrevision TKA	Completed

### 2.3. Operative Management and Hospital Course

An orthopedic consultation was obtained, and on May 28, the patient underwent a right knee irrigationand debridement(I&D) procedure, along with another polyethylene exchange, as part of a planned two‐step revision. A wound vacuum‐assisted closure (VAC) device was also placed during surgery. Purulent material was encountered intraoperatively. Five tissue specimens were sent for aerobic, anaerobic, fungal, acid‐fast bacilli (AFB), and miscellaneous cultures, all of which ultimately showed no growth.

On May 30, a second right knee debridement was set to be performed due to continued drainage. Before the surgery, she had another bout of left knee pain with +3 effusion noted, which was aspirated intraoperatively and found to contain purulent material, prompting I&D and VAC placement with wound cultures taken, which were negative. The right knee was also found to have purulent material and more diseased tissue. Absorbable antibiotic beads were placed in the right knee, and the incision was closed with an incision VAC placed. A second I&D of the left knee was performed on June 1 for continued infection control, although no gross infection was visualized, and a Hemovac drain was placed.

Throughout her admission, she continued to develop intermittent joint symptoms involving the left shoulder, right ankle, back, and right wrist. Her left shoulder was aspirated on May 31, yielding no fluid. MRI was discussed, but the patient declined. On June 5, she was scheduled for right knee antibiotic spacer placement; however, due to intense right ankle pain with warmth and erythema that morning, an ankle aspiration was performed intraoperatively and revealed purulence. An anteromedial ankle arthrotomy and I&D were performed, and the right knee procedure was postponed for a later date. Cultures from the ankle were also negative.

On June 6, the patient developed intermittent back and neck pain. Cervical, thoracic, and lumbar spine MRIs were performed, demonstrating a right L4–L5 facet with signal, surrounding edema, and enhancement consistent with septic arthritis, and a right L4–L5 lesion concerning for a small epidural or facet abscess (Figure 1). Neurointerventional radiology and neurosurgery were consulted, but interventional treatment was deferred due to the small lesion size (16 x 13 mm), and it was recommended that the MRI be repeated in a week. Around the same time, the patient reported experiencing pain and swelling in the right wrist. A bedside aspiration yielded scant, bloody fluid. The sample was insufficient for cell count, and her symptoms resolved without the need for surgical intervention.

**Figure 1:**
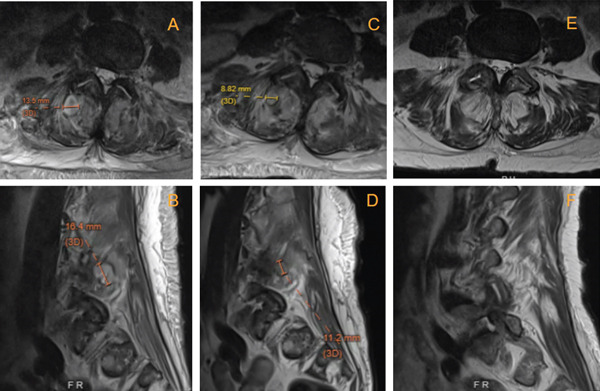
MRI lumbar spine timeline demonstrating right L4–L5 facet septic arthritis with associated paraspinal abscess and interval follow‐up. (A, B) (June 6,2025): (A) Axial and (B) sagittal T2‐weighted images show a spherical 16.4 × 13.5 mm hyperintense lesion posterior to the right L4–L5 facet articulation (arrows), surrounded by mild edema. Increased STIR signal and enhancement of the right facet articulation are also present, consistent with septic arthritis. (C, D) (June 13, 2025): (C) Axial and (D) sagittal T2‐weighted images redemonstrate right L4–L5 facet septic arthritis with a smaller adjacent abscess measuring 11.2 × 8.82 mm. (E, F) (July 18, 2025): (E) Axial and (F) sagittal T2‐weighted images show a persistent abnormal signal in the right facet articulation, compatible with septic arthritis, and near‐complete resolution of the paraspinal abscess in the right multifidus muscle belly posterior to the facet joint.

Given the persistent symptoms and new spinal findings, and with prior respiratory PCR positivity for *M. pneumoniae*, doxycycline was reinitiated empirically after finishing her last dose on May 30 while awaiting metagenomic next‐generation sequencing (mNGS) results. On June 10, plasma metagenomic cell‐free DNA sequencing (Karius test) returned positive for *M. pneumoniae*. At this point, ceftriaxone and vancomycin were discontinued, and doxycycline was continued as monotherapy, as per infectious disease recommendations, with a tentative plan to complete the course through at least July 22. The follow‐up lumbar MRI (Figure 1) on June 13 showed persistent facet septic arthritis but a smaller adjacent abscess (11 × 8 mm). A decision between the patient and care team was made to postpone further surgical intervention and continue antibiotic therapy to address potential additional foci of infection. The patient showed clinical improvement and was discharged on June 19, 24 days after admission, with revision surgery planned later in July.

### 2.4. Discharge and Follow‐Up

Follow‐up lumbar MRI (Figure 1) on July 18 demonstrated resolution of the previously noted 1 cm right multifidus abscess, though persistent septic arthritis changes remained at the right L4–L5 facet articulation. Revision of the right TKA was successfully performed on July 24 with cemented femoral and all‐tibia polyethylene components (Figure 2). Intraoperatively, samples were negative 14 days out. Doxycycline was discontinued postrevision given the absence of ongoing molecular evidence of active mycoplasma infection, and ertapenem was selected for standardized postoperative coverage following revision arthroplasty. Neurosurgery and infectious disease recommended continued nonoperative management of the facet infection, given clinical stability and interval improvement on MRI. She was discharged 4 days later and started on IV ertapenem (1 g daily) to complete a planned 6‐week course, given her history of prolonged antibiotic therapy. Subsequent lab testing and inflammatory markers show no abnormal findings, and the patient is progressing with full ROM of the right knee and no other joint complaints.

**Figure 2:**
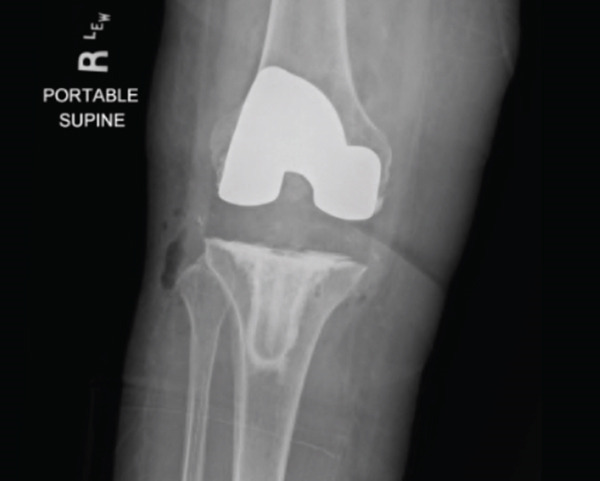
Anteroposterior of the right knee postrevision of knee replacement with all poly tibial insert.

Although plasma metagenomic sequencing suggested *M. pneumoniae* as the likely pathogen, broad‐spectrum antimicrobial therapy was reinitiated following revision arthroplasty. This decision reflected concern for potential polymicrobial infection, the prolonged and complex surgical course, extensive prior antibiotic exposure, and the risk of occult bacterial pathogens not detected by molecular testing. Empiric postoperative coverage is consistent with standard PJI management practices until intraoperative cultures remain negative and clinical stability is achieved, after which antimicrobial therapy can be safely narrowed.

## 3. Discussion

This case is notable for hematogenous spread of infection from a single infected right knee prosthesis to multiple native joints, including the contralateral knee, right ankle, lumbar facets, and possibly the right wrist and left shoulder, resulting in complex, multifocal septic arthritis. Risk factors contributing to disseminated disease likely included the patient′s underlying Waldenström macroglobulinemia and chronic anemia, both of which compromise host immune defense [1, 10, 11]. Waldenström macroglobulinemia is frequently associated with hypogammaglobulinemia, leading to impaired humoral immunity and increased susceptibility to opportunistic and atypical pathogens, including *M. pneumoniae* [10–12]. This immune dysfunction likely facilitated the hematogenous dissemination seen in our patient.

One of the main challenges in this case was the complete absence of growth on all cultures, despite clear purulence intraoperatively and systemic signs of infection. Culture‐negative PJI is reported in roughly 7%–11% of cases and is often linked to recent antibiotic exposure, fastidious organisms, or inadequate sampling [5, 13]. *M. pneumoniae* is particularly difficult to identify, as it lacks a peptidoglycan cell wall, which makes it inherently resistant to beta‐lactams and dependent on agents such as doxycycline or macrolides for treatment [14]. The organism is slow‐growing and fastidious, so culture results are frequently negative, especially in extrapulmonary disease [5, 6]. In practice, diagnosis relies on molecular testing, including PCR or mNGS [15, 16]. There is also some evidence that *M. pneumoniae* can form biofilms, which may help explain its ability to persist and evade immune clearance in chronic infections [17].

A turning point in this case came with the use of mNGS via the Karius test, which identified *M. pneumoniae* in the bloodstream. Plasma metagenomic sequencing was selected over synovial fluid PCR due to the patient′s multifocal disease, repeated negative intraoperative cultures, and concern for hematogenous dissemination. Synovial PCR would have required organism‐specific targets and repeat joint aspiration, whereas mNGS allowed unbiased pathogen detection from a single blood sample, particularly in the setting of prior antibiotic exposure. *M. pneumoniae* is a well‐known cause of atypical pneumonia, but it can also cause extrapulmonary manifestations, including septic arthritis, particularly in immunocompromised individuals [12, 14]. Mycoplasmal arthritis is often polyarticular and culture‐negative, necessitating the use of molecular techniques for detection. This case reinforces the growing recognition that *M. pneumoniae* should be considered in the differential diagnosis for disseminated or culture‐negative septic arthritis.

A distinctive feature of this case was the involvement of the right L4–L5 spinal facet joint, with imaging also revealing a small adjacent right paraspinal abscess. Septic arthritis of the facet joints (SAFJ) is rare, representing fewer than 5% of spinal infections, and is most often caused by *S. aureus*. Patients typically present with acute back pain and fever, and the lumbar spine is the most frequently affected region [18–22]. Multifocal disease is unusual, but hematogenous spread remains the most common route of infection [19, 21]. In this patient, the decision to pursue nonoperative management was driven by the small size of the abscess (16 × 13 mm), the absence of neurological compromise, a stable clinical status, and her steady response to antimicrobial therapy. Prior reports have noted that conservative treatment can be reasonable in carefully selected patients, particularly when abscesses are limited in size and close monitoring with imaging is feasible [18–22].

To our knowledge, there are no prior reports in the literature of spinal facet septic arthritis caused by *M. pneumoniae*, nor has facet involvement been documented as part of disseminated infection originating from a prosthetic joint [5, 6]. This adds to the clinical novelty of the case and underscores the potential for atypical presentations of mycoplasma infections, particularly in immunocompromised hosts.

The clinical utility of plasma cell‐free DNA sequencing, such as Karius testing used in this patient, and targeted multiplex PCR for diagnosing PJI has expanded significantly in recent years, with a meta‐analysis showing pooled sensitivity and specificity of 93% and 95%, respectively [15, 23, 24]. Compared with targeted PCR assays, mNGS offers broad, hypothesis‐free pathogen detection but at higher cost and longer turnaround time, particularly when sequencing and bioinformatic analysis are performed at reference laboratories [25, 26]. PCR assays are generally less expensive, have shorter turnaround times (often hours), and demonstrate high analytic specificity, but they require prior suspicion of the causative organism and may fail to detect unexpected or fastidious pathogens [27]. Reported diagnostic performance of mNGS in PJI demonstrates pooled sensitivity and specificity approaching 90%–95%, whereas PCR performance varies by assay design and organism target, with consistently high specificity but more variable sensitivity [23, 24, 27, 28]. Accordingly, these modalities should be viewed as complementary, with test selection guided by clinical context, pretest probability, and resource availability. In osteoarticular infections, mNGS demonstrates higher sensitivity than blood culture and improves diagnostic yield, particularly in chronic or indolent infections and in patients with prior antibiotic exposure, including infections caused by fastidious organisms such as *Mycoplasma*, *Cutibacterium*, and fungi [26, 29–31]. Although not yet standard in all institutions, these technologies are emerging as valuable adjuncts in the evaluation of complex PJI workups.

## 4. Limitations

Several limitations warrant acknowledgment. Organism‐specific molecular testing for *M. pneumoniae* was not performed on synovial fluid or intraoperative specimens, and universal PCR or 16S rRNA sequencing was not obtained from operative samples. As a result, attribution of *M. pneumoniae* as the causative pathogen is presumptive and based on concordant respiratory PCR positivity, plasma cell‐free DNA sequencing, a compatible clinical syndrome, and clinical response to targeted antimicrobial therapy. Earlier implementation of synovial fluid PCR or broad‐range molecular testing during the initial culture‐negative presentations may have expedited pathogen identification and potentially reduced the need for repeated surgical interventions and prolonged empiric antimicrobial therapy.

## 5. Conclusion

This case demonstrates a rare instance of hematogenous spread from a prosthetic knee infection to multiple native joints in an immunocompromised patient. Standard cultures remained negative, but advanced molecular testing identified *M. pneumoniae* as the likely pathogen, allowing for targeted antimicrobial therapy and avoidance of additional surgical morbidity. The patient′s multifocal infection required repeated debridements, close coordination between surgical and medical teams, and careful reconsideration of operative plans as her condition evolved.

This case reinforces the need for vigilance when evaluating prosthetic joint patients with systemic illness and new joint symptoms, even in the setting of negative cultures. Early infectious disease involvement, awareness of atypical pathogens, and the use of molecular diagnostics such as metagenomic sequencing can be critical in establishing a diagnosis and guiding therapy. More broadly, it highlights how individualized, multidisciplinary management is essential in complex, culture‐negative, and disseminated joint infections.

## Funding

No funding was received for this manuscript.

## Consent

Consent was obtained and a separate consent form filled out by the patient has been submitted.

## Conflicts of Interest

The authors declare no conflicts of interest.

## Data Availability

Data sharing is not applicable to this article as no datasets were generated or analyzed during the current study.
